# Histopathological assessment of inflammation and expression of inflammatory markers in patients with ketamine-induced cystitis

**DOI:** 10.3892/mmr.2014.3110

**Published:** 2014-12-18

**Authors:** HSIN-CHUNG LIN, HERNG-SHENG LEE, TZONG-SHI CHIUEH, YU-CHIEH LIN, HSIN-AN LIN, YU-CHUN LIN, TAI-LUNG CHA, EN MENG

**Affiliations:** 1 Department of Pathology, Tri-Service General Hospital, National Defense Medical Center, Taipei 11490, Taiwan, R.O.C.; 2 Department of Pathology, Taoyuan Armed Forces General Hospital, Taoyuan 32551, Taiwan, R.O.C.; 3 Division of Infection, Department of Medicine, Tri-Service General Hospital, National Defense Medical Center, Taipei 11490, Taiwan, R.O.C.; 4 Division of Infection, Department of Medicine, Tri-Service General Hospital SongShan Branch, Taipei 10581, Taiwan, R.O.C.; 5 Division of Urology, Department of Surgery, Tri-Service General Hospital, National Defense Medical Center, Taipei 11490, Taiwan, R.O.C.

**Keywords:** ketamine, cystitis, inflammation, cyclooxygenase-2, nitric oxide synthase, matrix metallopeptidase 9, ribosomal protein S6

## Abstract

The aim of the current study was to evaluate the histopathological features of inflammation and the expression levels of inflammatory markers in tissue samples from patients with ketamine-induced cystitis. Bladder biopsy samples for histological analysis were obtained from 23 patients (18 men and 5 women) with a self-reported history of ketamine use and who were treated for cystitis at the Tri-Service General Hospital of Taipei, Taiwan. Immunohistochemical staining for cyclooxygenase-2 (COX-2), inducible nitric oxide synthase (iNOS), matrix metallopeptidase-9 (MMP-9), mammalian target of rapamycin (mTOR), and phosphorylated 40S ribosomal protein S6 (Phos-S6) was performed. The results revealed urothelial atypia in all patients, and intravascular eosinophil accumulation in 22 patients. Histopathological features included denuded urothelial mucosa, ulceration, collagen deposition, smooth muscle degeneration and vessel proliferation. Tissue samples were immunopositive for all of the inflammation markers, including the urothelium, vessel walls, and smooth muscle. COX-2 staining revealed a significant difference between the inflammatory levels in the urothelium and smooth muscle, and iNOS staining differed significantly between inflammatory levels in smooth muscle (p=0.029). A positive correlation was observed between the percentage of Phos-S6-positive cells and the levels of inflammation in the urothelium. These results add to the descriptive literature on the histopathological aspects of ketamine-induced cystitis, emphasizing the inflammatory nature and a possible role for proteins such as COX-2, iNOS and Phos-S6 in the degree of inflammation.

## Introduction

Ketamine is a phencyclidine derivative first used as an intravenous anesthetic agent in 1965 (1) and approved by the US Food and Drug Administration in 1970 for use in producing analgesia and amnesia, with rapid recovery (2,3). It is predominantly an N-methyl-D-aspartate (NMDA) receptor antagonist, producing a state that is similar to catalepsy, termed dissociative anesthesia, in which sensory inputs appear to reach the cortical sensory areas but are not perceived, owing to suppression of association areas (4). In addition, ketamine affects non-NMDA glutamine receptors, as well as opioid, nicotinic, monoaminergic and muscarinic cholinergic receptors (5). While it is used medically as an anesthetic agent, the use of ketamine as a recreational drug in Taiwan has increased over the last 10 years, and it is emerging as an increasingly popular choice among young drug users, particularly in dance clubs (6,7). There is a prevailing misconception among this population that as a short-acting psychotropic agent, ketamine is not as harmful as other drugs, for example heroin, and that it has a broad margin of safety and a low potential for dependence. However, it has been reported that the majority of ketamine abusers (79%) display features of physiological dependence following a year of regular ketamine abuse, and 54% reported withdrawal symptoms on termination of use (8).

Ketamine abuse can also lead to severe lower urinary tract symptoms, complicated by decreased bladder capacity and hematuria. Ketamine-associated cystitis was first reported in case reports by Chu *et al* (9) and Shahani *et al* (10) in 2007 and was recently comprehensively reviewed by Wei *et al* (11). While the incidence of ketamine-associated lower urinary tract symptoms is difficult to assess, it is thought to be at least 30% among abusers (12). It has been suggested that the presence of ketamine and its active metabolites, including norketamine and dehydronorketamine, in the urine may damage the urinary tract mucosa (10). A recent study in rats reported that ketamine treatment results in bladder hyperactivity, along with ulcerated urothelium and mononuclear cell infiltration (13). These alterations were accompanied by significant increases in the expression levels of cyclooxygenase-2 (COX-2) and two nitric oxide synthase (NOS) isoforms [inducible NOS (iNOS) and endothelial NOS (eNOS)], which were determined by western blot analysis, in addition to a significant increase in the number of COX-2-positive cells, as determined by immunohistochemistry (13). The authors suggested that these pathological changes, together with the upregulation of inflammatory proteins, may have an important role in ketamine-induced ulcerative cystitis in rats. The aim of the present study was to assess the histopathological features and the degree of inflammation in the bladder urothelium, vessel walls, and smooth muscle of patients with ketamine-induced cystitis, to determine if the expression levels of inflammatory markers, such as those described above (13), had correlated with the degree of inflammation.

## Materials and methods

### Patients and sample collection

This study was approved by the Institutional Review Board of the Tri-Service General Hospital (Taipei, Taiwan). A total of 23 patients at the Tri-Service General Hospital with a self-reported history of ketamine abuse and a confirmed diagnosis of cystitis were included in this retrospective study. Each patient presented with lower urinary tract symptoms such as urgency, nocturia and frequency. Urine and blood samples were collected from each patient for analysis. The urine test panel included strip glucose, urine protein, urine bilirubin, urobilinogen, pH, occult blood, acetone in urine, strip white blood cells (WBCs), nitrite, clarity, specific gravity and color and sediments [urine red blood cells (RBCs), urine WBCs, epithelial cells, urine casts, bacteria, crystals, yeast, spermatozoa, *Trichomonas*, dysmorphic RBCs and mucus]. Blood analysis included WBC count, RBC count, hemoglobin, hematocrit, mean corpuscular volume (MCV), mean corpuscular hemoglobin (MCH), mean corpuscular hemoglobin concentration (MCHC = MCH/MCV), platelet count and differential count (neutrophils, lymphocytes, monocytes, eosinophils and basophils). Paraffin-embedded urothelial tissues were obtained by urothelial biopsy 1 week following the urine and blood sample collection. Marked urothelial atypia with nuclear enlargement was evident in association with urothelial ulceration. Normal urothelial tissue was collected from nearby tissue without symptoms of inflammation. All participants provided written informed consent. The institutional review boards of the hospital approved the study protocol.

### Assessment of inflammation

Paraffinized sections (0.6 μm) from each patient (3–10 sections/patient) were stained with Gill’s hematoxylin V (MUTO, Tokyo, Japan) and 1% eosin alcohol solution (MUTO; H&E). The degree of inflammation was assessed according to a semi-quantitative scale. H&E-stained slides were examined under a light microscope (Olympus BX51; Olympus, Tokyo, Japan) in a double-blind manner by two histopathologists. Inflammation was scored as follows: mild inflammation, <5 mononuclear inflammatory cells in a 10×10 grid (area, 0.25 mm^2^; magnification, ×200); moderate inflammation, mononuclear inflammatory cells scattered throughout the tissue but background stromal connective tissue clearly visible; severe inflammation, mononuclear inflammatory cells densely infiltrating the tissues. The degree of inflammation in each case was assessed throughout the specimen. Although lymphoid follicles with germinal centers were encountered, these were not assessed.

### Immunohistochemical staining

Paraffinized sections (0.6 μm) were generated for immunohistochemistry from patients (3–10 sections/patient) with sufficient specimens from the urothelial biopsy. When >1 block was available, we used the same block used for histopathologic diagnosis. Sections were mounted on silanized glass slides, stored in the dark at 58°C, and subjected to immunohistochemical analysis within 1 week. Following paraffin removal and rehydration, the sections were subjected to antigen retrieval by microwave heating in 10 mM citrate buffer, pH 6.0 (Merck Eurolab, Copenhagen, Denmark) twice, for 5 min each time. Endogenous peroxidase activity was blocked by incubation in 5% H_2_O_2_ in distilled water for 20 min at room temperature, followed by 30 min incubation at room temperature with primary antibodies targeting COX-2 (monoclonal, anti-mouse; Thermo Labsystems, Santa Rosa, CA, USA), iNOS (monoclonal, anti-mouse; Thermo Labsystems), and matrix metallopeptidase-9 (MMP-9; monoclonal, anti-mouse; Abcam, Cambridge, UK) (14), as well as cancer-related markers, including mammalian target of rapamycin (mTOR; monoclonal, anti-mouse; Thermo, Rockford, IL, USA) and phosphorylated 40S ribosomal protein S6 (Phos-S6; monoclonal, anti-mouse; Immunotech, Marseille, France). All of the antibodies were diluted to 1:200 in antibody diluent (Dako, Glostrup, Denmark) (15–17). Sections were subsequently incubated with secondary antibody [EnVision™ FLEX/HRP + Rabbit/Mouse (LINKER)] for 20 min at room temperature. Intervening washes were performed in EnVision FLEX wash buffer for 5 min each. For COX-2, antigen-antibody complex was visualized with 3,3′-diaminobenzendine (EnVision FLEX DAB + Chromogen). For iNOS, MMP-9, mTOR and Phos-S6, the antigen-antibody complex was visualized using the 3-amino-9-ethylcarbazole (AEC) color system (MUTO), and sections were counterstained with Mayer’s hematoxylin (MUTO). The stained sections without primary antibodies were used as a negative control. Slides were coverslipped with Glycergel Mounting Medium (Dako) and examined under an Olympus BX51 light microscope in a double-blind manner by two histopathologists. The staining intensity of the urothelium, vessel walls and smooth muscle was scored as follows: 0, no staining; 1, mild staining; 2, moderate staining; 3, intense staining. In addition the percentage of positive cells within a 10 × 10 cm grid (area, 0.25 mm^2^; magnification, ×200) was calculated (18). The percentage of positive cells was counted regardless of staining intensity.

### Statistical analysis

Statistical analysis was performed using SPSS statistics software version 15.0 (SPSS, Inc., Chicago, IL, USA). The patient demographic and clinical characteristics are presented as the mean ± standard deviation (SD) for continuous data and n (%) for categorical data. Morphological data are presented as n (%) by inflammatory stage. The differences among the inflammatory stages were compared using Fisher’s exact test. Spearman correlation analysis was performed to identify correlations between the morphologic data and inflammatory stage. Results are presented as the coefficient of correlation (r) and the corresponding P-value. Statistical assessments were two-tailed and significance was set at P<0.05.

## Results

**Figure 1:**
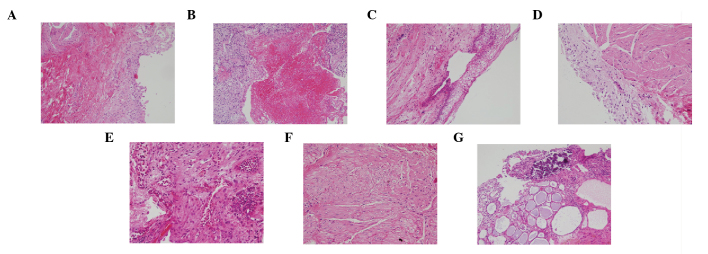
Morphology of the urinary bladder in ketamine-induced cystitis. Urothelial mucosa in ketamine-induced cystitis stained with hematoxylin and eosin demonstrating (A) denuded, (B) ulcerated, (C) clear change with a suburothelial blister and (D) urothelial nuclear atypical morphologies. The stroma of the urinary bladder revealed (A) collagen deposition, (E) intravascular eosinophils acumination, (F) smooth muscle degeneration and (G) calcification. In addition, (G) lymphatic duct proliferation was noted in only one patient. (magnification, ×200).

A total of 23 subjects (18 males and 5 females) were analyzed in this study. Demographic and clinical characteristics are summarized in Table I. The average age was 21.8±3.7 years (mean ± SD). Duration of ketamine usage ranged from 2 to 6 years, however, there were 12 patients for whom this information was not available. The morphology of the bladder in ketamine-induced cystitis was highlighted by staining with H&E, as shown in Fig. 1. Mucosal histologic features of ketamine-associated cystitis included the following in variable numbers of patients (Fig. 1): denuded urothelial mucosa with a thinner layer of epithelial cells, ulceration, changes in clarity, increased collagen deposition, smooth muscle degeneration in the stromal tissue, vessel proliferation, calcification, and inflammation with variable numbers of neutrophils, eosinophils, and mast cells. Inflammation was mild for 2 patients (8.7%), moderate for 15 (65.2%) and severe for 6 (26.1%). All of the patients received a diagnosis of urothelial atypia, and the majority (22/23) showed intravascular eosinophil accumulation. In addition, lymphatic duct proliferation was noted in only one patient.

### Urine analysis

Seven patients showed no protein in the urine, two showed equivocal results, five showed 1^+^ proteinuria, eight showed 2^+^, and one showed 3^+^. Only one patient had urine bilirubin. The urine pH range was 5.5–7.5. Testing for occult blood was negative in seven patients, equivocal in two, 1^+^ in one, 2^+^ in three, and 3^+^ in ten. The results of testing for RBCs were <10 in nine patients, 10–100 in four and >100 in ten. The results of testing for WBCs were <10 in 13 patients, 10 to 100 in seven, and >100 in three. Testing for bacteria was negative in 17 patients, equivocal in one, 1^+^ in three and 2^+^ in two.

### Blood analysis leukocytosis was present in three patients, and six were anemic

All 23 patients had a normal platelet range. Four patients showed neutrophilia, one showed neutropenia, and seven showed lymphocytopenia. Five patients showed an increase in the levels of monocytes, and one showed a decrease in the levels of monocytes. One patient showed eosinophilia.

### Immunohistochemical staining for inflammation markers

**Figure 2:**
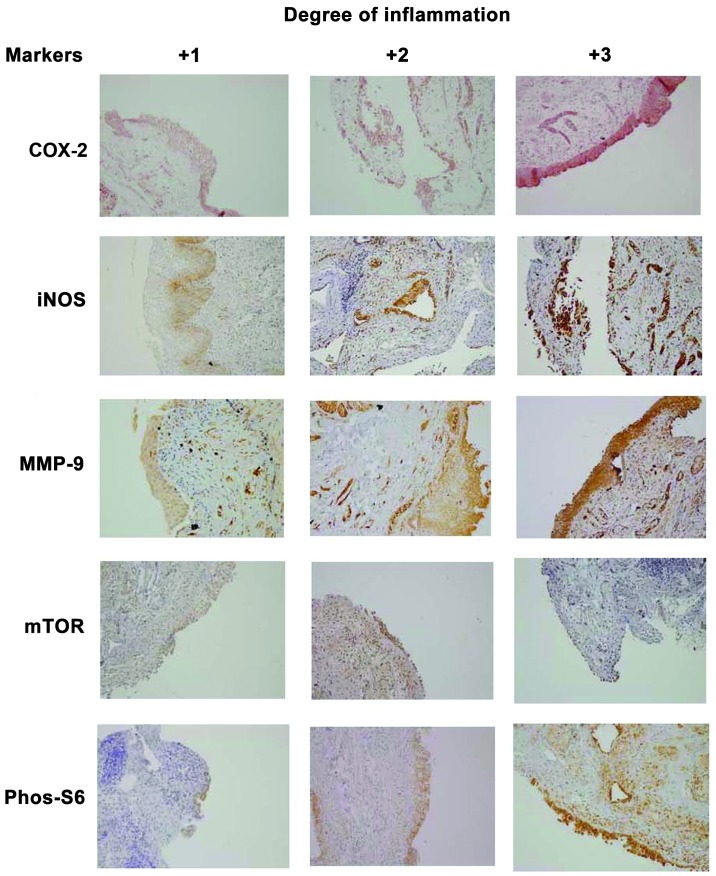
Immunohistochemical staining of cyclooxygenase-2 (COX-2), inducible nitric oxide synthase (iNOS), matrix metallopeptidase-9 (MMP-9), mammalian target of rapamycin (mTOR) and phosphorylated 40S ribosomal protein S6 (Phos-S6) in the urothelium with different degrees of inflammation (magnification, ×200).

Immunostaining in tissues with different degrees of inflammation (mild, moderate and severe) revealed staining for all five of the inflammation markers (Fig. 2). Quantification of the immunohistochemical staining of sections from certain patients is presented in Tables II and III. Table II displays immunohistochemical staining intensity (0–3) by inflammation level (mild, moderate and severe) in the urothelium, vessel walls, and smooth muscle. COX-2 staining differed significantly between the degrees of inflammation (mild, moderate and severe) in the urothelium and smooth muscle (P=0.027 urothelium; P=0.020 smooth muscle). In the urothelium, for example, only one specimen stained 3^+^ and one specimen stained 2^+^ for COX-2 in the tissue with mild inflammation. However, all four specimens from the tissue with severe inflammation stained 3^+^ for COX-2 (Table II). In addition, iNOS staining differed significantly between the degrees of inflammation in smooth muscle (P=0.029). No significant difference was found between the degrees of inflammation for MMP-9, Phos-S6 or mTOR staining.

Table III shows the percentage of immunostaining positive cells by degree of inflammation. Results of the Spearman correlation analysis indicated a positive correlation between the percentage of Phos-S6-positive cells and the degree of inflammation in the urothelium (r=0.815; P=0.001) but no in other tissues. No section from normal tissue and negative control showed immunostaining positive cells.

## Discussion

The aim of the present study was to evaluate the histopathological features and inflammation status of samples from patients with ketamine-induced cystitis to determine if markers of inflammation were expressed. The results revealed histopathological features consistent with those previously reported (7,9,10–12), including marked intravascular eosinophil accumulation and variable denuded urothelial mucosa and ulceration; the majority of patients (65.2%) showed moderate inflammation, with the remainder showing severe (26.1%) or mild (8.7%) inflammation. Samples showed positive immunostaining for all 5 of the inflammation markers assessed, with COX-2 staining differing significantly between the three degrees of inflammation in the urothelium and smooth muscle, and iNOS staining differing significantly with inflammation level in smooth muscle.

The mechanism of ketamine-induced ulcerative cystitis remains to be elucidated. Results from animal models (13) and the pathological data of the current study suggest that the urothelium may play a role in the pathogenesis of ketamine-induced cystitis. The etiology of ketamine-induced cystitis may be the direct toxic effect of ketamine and its active metabolite on the bladder mucosa (7). Ketamine induces significant submucosal hemorrhage, enhanced macrophage infiltration, ulceration, reduced urothelium thickness and the degeneration of smooth muscle. These pathological changes may be the cause of the ketamine-induced expression of COX-2, iNOS and MMP-9 in the urothelium, vessel wall and smooth muscle. The present results, along with those in rats (13), suggest that inflammatory markers, including COX-2 and iNOS, may be involved, either as a result of ketamine-induced cystitis or inducing it. Notably, an increase in the expression of COX-2 and iNOS has been reported in response to cyclophosphamide-induced cystitis in rats (19,20). Matrix metalloproteinase (MMP)-2 and MMP-9, collectively known as the gelatinases, are closely associated with inflammatory and infectious diseases in a number of organs (21). However, the current study did not find a correlation between the MMP-9 expression and inflammation levels in tissues of ketamine-induced cystitis.

Notably, the histopathological features of ketamine-induced cystitis in this study showed a marked intravascular eosinophil accumulation, suggesting that the inflammatory response may begin from the blood vessels in the bladder. However, significant differences in the COX-2 and iNOS staining intensity as well as % immunostaining positive cells were not observed between tissues from three degrees of inflammation. It has been suggested that other inflammatory mediators induced by ketamine may be involved in the induction of intravascular eosinophil accumulation.

The results of the present study indicated a positive correlation between the percentage of Phos-S6-positive cells and inflammation level in the urothelium. The expression of mTOR and mTOR pathway members, such as Phos-S6, have been reported in bladder carcinoma (15–17,22). Oxley *et al* (23) have reported that ketamine-induced cystitis is a mimic of carcinoma *in situ*. The results of the current study confirmed their observations that the markers of bladder carcinoma may be observed in the tissues of patients with ketamine-induced cystitis. However, it is unclear at this time why there was a positive correlation between the percentage of Phos-S6-positive cells in the urothelium and the degree of inflammation.

Limitations of the present retrospective study include the fact that no samples were analyzed from control subjects (e.g. non-ulcerative cystitis or healthy tissues from non-abusers) due to the ethical reasons, and the fact that immunohistochemical staining was not performed for all subjects. In addition this study did not assess the ketamine level in urine samples, or the association between duration of ketamine usage and severity of ulcerative cystitis (severity of inflammation). The latter information was not available for 12 subjects. In conclusion, these results add to the descriptive literature on histopathologic aspects of ketamine-induced cystitis, emphasizing the inflammatory nature and a possible role for proteins such as COX-2, iNOS and Phos-S6. Ketamine and its active metabolites may have a direct toxic effect on the bladder mucosa; the observed histopathologic changes may induce or increase expression of COX-2, iNOS and Phos-S6 in the urothelium and smooth muscle.

## Figures and Tables

**Table I:** Demographic characteristics of the 23 subjects and the morphological features of their urinary bladder.

Characteristic	n (%)^a^
Age (years)^a^	21.8±3.7
Gender	
Male	18 (78.3)
Female	5 (21.7)
Denuded urothelial mucosa	
Yes	13 (56.5)
No	10 (43.5)
Ulceration	
Yes	10 (43.5)
No	13 (56.5)
Urothelial atypia	
Yes	23 (100)
No	0
Intravascular eosinophil accumulation	
Yes	22 (95.7)
No	1 (4.3)
Collagen deposition	
Yes	15 (65.2)
No	8 (34.8)
Smooth muscle degeneration	
Yes	7 (30.4)
No	16 (69.6)
Vessel proliferation	
Yes	12 (52.2)
No	11 (47.8)
Calcification	
Yes	3 (13.0)
No	20 (87.0)
Mucosal clear change	
Yes	2 (8.7)
No	21 (91.3)
Inflammatory stage	
Mild	2 (8.7)
Moderate	15 (65.2)
Severe	6 (26.1)

With the exception of age, which is presented as the mean ± standard deviation.

**Table II:** Immunostaining for inflammation markers and histologic assessment of inflammation in the urothelium, vessel wall and smooth muscle.

	Urothelium					Vessel wall					Smooth muscle				
			
		Inflammation					Inflammation					Inflammation			
									
Staining intensity^a^	No. of samples^b^	Mild (n=2)	Moderate (n=15)	Severe (n=6)	P-value	No. of Samples^b^	Mild (n=2)	Moderate (n=15)	Severe (n=6)	P-value	No. of Samples^b^	Mild (n=2)	Moderate (n=15)	Severe (n=6)	P-value
COX-2					0.027^c^					1.000					0.020^d^
0	0	0	0	0		1	0	1	0		1	0	1	0	
1	1	0	1	0		5	1	3	1		3	0	1	2	
2	6	1	5	0		4	0	3	1		6	1	1	4	
3	6	1	1	4		8	1	4	3		8	1	7	0	
MMP-9					0.626					0.384					0.335
0	0	0	0	0		1	0	1	0		1	0	1	0	
1	4	1	2	1		0	0	0	0		3	0	1	2	
2	2	0	2	0		6	1	2	3		9	1	5	3	
3	9	0	6	3		14	1	10	3		7	1	6	0	
iNOS					0.643					0.808					0.029^c^
0	0	0	0	0		3	1	2	0		3	1	1	1	
1	0	0	0	0		4	0	2	2		8	0	3	5	
2	2	0	2	0		4	0	2	2		7	1	6	0	
3	11	2	5	4		7	1	4	2		0	0	0	0	
Phos-S6					0.201					0.774					0.327
0	0	0	0	0		2	1	1	0		6	1	4	1	
1	2	1	1	0		0	0	0	0		3	1	0	2	
2	5	1	4	0		7	1	4	2		3	0	2	1	
3	5	0	2	3		4	0	2	2		0	0	0	0	
mTOR					1.000					0.778					0.335
0	0	0	0	0		2	1	1	0		0	0	0	0	
1	1	0	0	1		4	0	3	1		1	0	1	0	
2	5	1	2	2		5	1	2	2		5	0	2	3	
3	1	0	0	1		3	0	1	2		6	1	3	2	

a Staining intensity: 0, no staining; 1, mild staining; 2, moderate staining; 3, intense staining.

b Samples were not obtained for all 23 patients. Data are presented as n (%). Differences among inflammation levels were compared by the Fisher’s exact test.

c P<0.05 and

d P<0.01 dispersion of immunohistochemical staining intensity between mild, moderate and severe inflammation status, by pairwise comparison.

COX-2, cyclooxygenase-2; iNOS, inducible nitric oxide synthase; MMP-9, matrix metallopeptidase-9; mTOR, mammalian target of rapamycin; Phos-S6, phosphorylated 40S ribosomal protein S6.

**Table III:** Percentage of cells immunopositive for inflammation markers and histologic assessment of inflammation in the urothelium, vessel wall and smooth muscle.

		Urothelium				Vessel wall				Smooth muscle			
				
Marker	Inflammation	No. of samples^a^	% Positive cells^b^	r-value^c^	P-value	No. of samples^a^	% Positive cells^b^	r-value^c^	P-value	No. of % samples^a^	Positive cells^b^	r-value^c^	P-value
COX-2				0.194	0.526			0.049	0.851			−0.088	0.736
	Mild	2	80.0			2	40.0			2	50.0		
	Moderate	7	61.4			10	52.0			9	47.8		
	Severe	4	80.0			5	48.0			6	41.7		
MMP-9				0.209	0.454			−0.105	0.658			0.020	0.933
	Mild	1	90.0			2	45.0			2	55.0		
	Moderate	10	77.0			12	70.0			13	65.4		
	Severe	4	87.5			6	51.7			5	60.0		
iNOS				0.315	0.295			0.159	0.572			−0.002	0.994
	Mild	2	85.0			1	30.0			2	40.0		
	Moderate	7	74.3			8	50.0			10	39.0		
	Severe	4	87.5			6	51.7			5	36.0		
Phos-S6				0.815	0.001^d^			0.286	0.394			0.112	0.833
	Mild	2	20.0			4	67.5			1	10.0		
	Moderate	7	50.0			1	60.0			2	25.0		
	Severe	3	83.3			6	53.3			3	20.0		
mTOR				0.250	0.588			−0.263	0.409			0.020	0.951
	Mild	1	30.0			1	70.0			1	30.0		
	Moderate	2	60.0			6	70.0			6	41.7		
	Severe	4	65.0			5	46.0			5	39.0		

a Samples were not obtained for all 23 patients.

b Data are presented as the mean.

c Coefficient of correlation (r) was derived by the Spearman correlation analysis. A positive r-value indicates a positive correlation, and a negative r-value indicates a negative correlation.

d Significant correlation.

COX-2, cyclooxygenase 2; iNOS, inducible nitric oxide synthase; MMP-9, matrix metallopeptidase-9; mTOR, mammalian target of rapamycin; Phos-S6, phosphorylated 40S ribosomal protein S6.
